# Nanoparticles of Thiolated Xanthan Gum for the Oral Delivery of Miconazole Nitrate: In Vitro and In Vivo Evaluation

**DOI:** 10.3390/pharmaceutics16020225

**Published:** 2024-02-04

**Authors:** Nader I. Namazi, Hamad Alrbyawi, Abdulkareem Ali Alanezi, Afaf F Almuqati, Anwar Shams, Hany S. M. Ali

**Affiliations:** 1 Department of Pharmaceutics and Pharmaceutical Industries, College of Pharmacy, Taibah University, Madinah 41477, Saudi Arabia; hrbyawi@taibahu.edu.sa (H.A.); hsali@taibahu.edu.sa (H.S.M.A.); 2 Department of Pharmaceutics, College of Pharmacy, University of Hafr Al-Batin, Hafr Al-Batin 31991, Saudi Arabia; aalanezi@uhb.edu.sa; 3 Department of Pharmaceutical Chemistry, College of Pharmacy, University of Hafr Al-Batin, Hafr Al-Batin 31991, Saudi Arabia; aalmaqati@uhb.edu.sa; 4 Department of Pharmacology, College of Medicine, Taif University, Taif 21944, Saudi Arabia; a.shams@tu.edu.sa; 5 Centre of Biomedical Sciences Research (CBSR), Deanship of Scientific Research, Taif University, Taif 21974, Saudi Arabia; 6 High Altitude Research Center, Taif University, Taif 21944, Saudi Arabia; 7 Department of Pharmaceutics, Faculty of Pharmacy, Assiut University, Assiut 71526, Egypt

**Keywords:** xanthan gum, thiolation, nanoparticles, antifungal activity, pharmacokinetics

## Abstract

The objective of this research was to develop a mucoadhesive delivery system that improves permeation for the administration of poorly absorbed oral medications. Thiolation of xanthan gum (XGM) was carried out by esterification with mercaptobutyric acid. Fourier-transformed infrared spectroscopy was used to confirm thiol-derivatization. Using Ellman’s technique, it was revealed that the xanthan-mercaptobutyric acid conjugate had 4.7 mM of thiol groups in 2 mg/mL of polymeric solution. Using mucosa of sheep intestine, the mucoadhesive properties of XGM and thiolated xanthan gum (TXGM) nanoparticles were investigated and we found that TXGM had a longer bioadhesion time than XGM. The disulfide link that forms between mucus and thiolated XGM explains why it has better mucoadhesive properties than XGM. A study on in vitro miconazole (MCZ) release using phosphate buffer (pH 6.8) found that TXGM nanoparticles released MCZ more steadily than MCZ dispersion did. A 1-fold increase in the permeation of MCZ was observed from nanoparticles using albino rat intestine compared to MCZ. Albino rats were used to test the pharmacokinetics of MCZ, and the results showed a 4.5-fold increase in bioavailability. In conclusion, the thiolation of XGM enhances its bioavailability, controlled release of MCZ for a long period of time, and mucoadhesive activity.

## 1. Introduction

The imidazole group’s wide-spectrum antifungal drug MCZ is frequently used to treat oral candidiasis, an opportunistic fungal infection of the oral cavity brought on by an overabundance of Candida species [1]. Of them, the most common species found in oral cavity isolates from both healthy and sick people is *Candida albicans*. MCZ is typically taken as an oral gel to treat oral candidiasis. Unfortunately, the limited aqueous solubility of this antifungal drug may limit its efficacy when preparing solid formulations for oral administration [2]. The creation of MCZ-loaded nano carrier systems, such nanoparticles, is one possible way to get over this hurdle [3]. The encapsulation of drugs into nanoparticles has been demonstrated to enhance the antifungal efficacy of these systems, making them ideal carriers for the administration of weakly water soluble antifungals such as clotrimazole, MCZ nitrate, and amphotericin B.

One kind of extracellular polysaccharide that is secreted by the bacteria *Xanthomonas campestris* is called XGM. A terminal D-mannose unit is connected to a pyruvate group, D-glucose, D-mannose, and D-glucoronic acid in a ratio of 2:2:1. The structure is made up of a linear D-glucose backbone connected to a trisaccharide side chain containing D-mannose with an acetyl group, D-glucuronic acid. XGM is resistant to enzymatic degradation and is very stable over a broad pH and temperature range [4]. It is widely utilized in the food and pharmaceutical industries because of its non-toxic and biocompatible qualities. Although the mucoadhesion duration is quite short, XGM is widely employed in mucoadhesive drug delivery systems [5]. Due to less mucoadhesion time, the availability of the drug for absorption is low which ultimately lowers the drug’s bioavailability. In order to improve the mucoadhesion qualities, mercaptobutyric acid was used to thiolate the XGM in this work.

Many studies have been conducted recently on the development of bioadhesive/mucoadhesive controlled release systems [6]. It is commonly acknowledged that raising a drug’s viscosity to impede clearance and extending the duration of contact through mucoadhesive interactions may increase the drug’s, or any therapeutic’s, bioavailability [7]. For these kinds of applications, polymeric enhancers or mucoadhesive polymers might be a good option. They have use in the oral distribution of medications because they can improve permeability [8]. One useful technique for drug immobilizationor localization is mucoadhesion, which is the binding of a synthetic or natural polymer to a biological surface. This is an essential component in controlled drug delivery. Although mucoadhesion is not a novel topic, a spike in curiosity has occurred in the use of mucoadhesive polymers for medication administration in recent years [8,9]. Recently there has been a lot of work on focusing a medication or formulation in one area of the body over extended periods of time [10]. Medications that are absorbed via the mucosal lining of tissues can enter the blood stream directly and not be rendered inactive by enzymatic degradation in the gastrointestinal tract, which is why this is necessary not only for targeting medications but also for the improved regulation of systemic drug distribution [8]. These polymers’ mucoadhesive qualities or the mucoadhesion mechanism can be described by the ionic interactions and production of non-covalent bonds, such as hydrogen bonds, between the mucoadhesive polymer and the mucus layer. Previous research on polymers has involved thiolation with mercaptopropionic and thioglycolic acid, which, when compared to the original polysaccharide, demonstrated good mucoadhesion capabilities [11,12].

In this research work, XGM was thiolated with mercaptobutyric acid for the enhancement of mucoadhesion. Mercaptobutyric acid has excellent optical properties and is safe for humans due to its use in foods and beverages. Mercaptobutyric acid has an appropriate chain length [13] and improves the mucoadhesive properties of XGM by incorporating the thiol group. Due to the presence of a sulfhydryl group in mucin, TXGM forms a disulfide bond which enhances mucoadhesion strength. According to our best knowledge from the literature search, mercaptobutyric acid is being used for the first time for the thiolation of xanthan gum to enhance the mucoadhesion time. The developed nanoparticles were characterized by Fourier transforms infrared spectroscopy (FTIR), differential scanning calorimetry (DSC), thermogravimetric analysis (TGA), X-ray diffraction (XRD) and scanning electron microscopy (SEM). The nanoparticles were also evaluated for drug release, stability, antifungal activity and pharmacokinetics of MCZ using albino rats after oral delivery.

## 2. Materials and Methods

### 2.1. Materials

MCZ was gifted by Saffron Pharmaceuticals, Pvt. Ltd., Faisalabad, Pakistan. Xanthan gum (XGM), mercaptobutyric acid, methanol and HCl were purchased from Sigma Aldrich Gmbh, Darmstadt, Germany. Trypsin and potassium dihydrogen phosphate were purchased from Merck, Darmstadt, Germany.

### 2.2. Thiolation of XGM

Using mercaptobutyric acid (MBA) and a catalytic quantity of hydrochloric acid, the esterification reaction was utilized to thiol-functionalize XGM. Two moles of MBA were added to each hydroxyl group in one mole of XGM to carry out the reaction. A catalytic amount of HCl (7 N) and MBA (4.2 mL) were added to 250 mL of hot water to dissolve 8 g of XGM. For 150 min, the reaction mixture was refluxed at 80 °C. Methanol was added to the reaction mixture above, allowing it to cool and precipitate. The resulting precipitates were then dried in an oven set to 50 °C after being rinsed with methanol twice [12].

### 2.3. ^1^H NMR Analysis

^1^H NMR spectra of XGM and TXGM were obtained by dissolving them in deuterated NMR solvents at 400-MHz spectrometer using a ^1^H NMR spectrometer (Bruker Avance III) Harwell, UK.

### 2.4. Determination of Thiol Contents

Ellman’s reagent [DTNB] was used in a colorimetric process to quantify the contents of thiol of TXGM [14]. The free thiol groups of TXGM react with DTNB in this reaction to generate a colored conjugate, whose absorbance may be measured at 412 nm using a UV/vis spectrometer (UNICAM 8700 series). To put it briefly, 500 µL of 0.5 M phosphate buffer pH 8.0 was used to hydrate 0.5 mg of each conjugate. The samples absorbance was evaluated at 412 nm following a 2-h incubation period at 25 °C with 500 µL of Ellman’s reagent (0.03% (*w*/*v*) DTNB in 0.5 M phosphate buffer pH 8.0). With the help of linearity curve resulting from the sulfhydryl group measurement of solutions containing different amounts of cysteine (0.003–0.05 mg/mL), the contents of thiol groups in TXGM were determined. With the help of linearity curve resulting from the sulfhydryl group measurement of solutions containing different amounts of cysteine holding a constant concentration of unmodified XGM (0.5 mg/mL), the quantity of thiol groups of TXGM was determined.

### 2.5. Preparation of Nanoparticles

Prepare 1% solution of previously dried xanthan gum powder that makes it 0.5 g of previously dried and processed thiolated xanthan gum in 50 mL of deionized water and stir for 30 min. Then prepare tri-sodium polyphosphate (STPP) solution with the concentration of 0.7 mg/mL and with the final volume of 50 mL. After 30 min add 50 mL of STPP solution in the xanthan gum solution drop wise and stir for about an hour. After 1 h, centrifuge the mixture for about 60 min at 6000 rpm. Separate the supernatant, dry the precipitates, and again grind it into a fine powder. Five formulations were prepared by changing the concentration of STPP shown in Table 1. The particle size was reduced by using a high pressure homogenizer (Camsdorfer Ufer 12 07749, Jena, Germany) at 23,000 rpm for 18 min with 3 intervals of 6 min and pausing for 1 min after every interval. Then centrifuge (De Novo Tech, 27AFUPN0722K1Z2, Pune, India) the mixture at 12,000 rpm for 15 min. After 15 min, separate the supernatant from the dried particles and grind it to fine powder [15].

### 2.6. Size and Surface Charge Analysis

Particle size and polydispersity index (PDI) were measured by diluting specimens in a polystyrene cuvette using double-distilled water [16]. Dynamic light scattering (DLS) was used to measure the samples at a temperature of 25 °C and with a scattering angle of 90° using a Beckman Coulter N5 Particle Size Analyzer (Beckman, Brea, CA, USA). In order to describe the size distribution, PDI was also measured. The particles’ zeta potential was measured at 25 °C using a Zetasizer (Malvern Instrument Ltd., Malvern, UK, ZS 90) after an appropriate dispersion was made using double-distilled, de-ionized water. The average particle size, PDI and zeta potential were obtained by repeating each sample three times (*n* = 3).

### 2.7. Loading and Entrapment Efficiency of MCZ

A precisely measured quantity of nanoparticles was mixed with 1 mL of phosphate buffer saline (pH 7.4). The addition of trypsin (0.1 mg/mg of NPs) degraded the particles in the solution [17]. After centrifuging the mixture and stirring it for the entire night, the drug concentration of the supernatant was examined (Equation (1)). All the measurements of % MCZ loading were triplicated (*n* = 3).

(1) $$\%\;MCZ\;loading=\frac{Amount\;of\;miconazole\;in\;nanoparticles}{Weight\;of\;nanoparticles}\times 100$$

A 1 mL sample was spun at 40,000 rpm for 20 min using a Beckman-Coulter Optima MAX-XP ultra-centrifuge (Beckman, Brea, CA, USA) in order to evaluate the MCZ entrapment in nanoparticles. The MCZ content in the supernatant was examined, and the entrapment efficiency (*n* = 3) was computed using the equation below:

(2) $$\%\;entrapment\;efficiency=\frac{Total\;amount\;of\;MCZ\;added-amount\;of\;MCZ\;in\;supernatant}{Total\;MCZ\;added}\times 100$$

### 2.8. Release of MCZ from Nanoparticles

MCZ-loaded nanoparticles containing 10 mg of the MCZ were placed inside the dialysis bags (dialyzing membrane-150, molecular weight cutoff 12,000–14,000 Dalton) in order to evaluate the MCZ release from the nanoparticles. Before use, the membrane was soaked in water for 30 min. The dialysis bag was incubated for 24 h at 37 ± 1 °C with constant stirring at 100 rpm in 900 mL of pH 6.8 phosphate buffer. To keep the sink condition, 2 mL of sample volume were removed and replaced with fresh buffer at predetermined intervals. Using HPLC, the amount of MCZ released in the dissolution media was measured [18]. The release of MCZ from all prepared nanoparticulate formulations was repeated six times (*n* = 6).

### 2.9. Characterization of Nanoparticles

#### 2.9.1. Fourier Transforms Infrared Spectroscopy (FTIR)

MCZ, XGM, TXGM, blank and MCZ-loaded nanoparticles werechecked for determination of thiol and other functional groups using ATR-FTIR Bruker, Alpha, Germany at 400–4000 cm^−1^ wavelength with a resolution of 1 cm^−1^.

#### 2.9.2. Differential Scanning Calorimetry (DSC)

A Mettler DSC 823 (Mettler-Toledo, Greifensee, Switzerland) was used to execute a DSC analysis of MCZ, XGM, TXGM, and lyophilized nanoparticles of NTG1. Weighed and tightly sealed, the milled samples were placed within an aluminum pan. Both the reference cell and the sample were evacuated with the flow of nitrogen during the measurement. The samples were put inside the cell of the sample holder and heated to 500 °C, with a 10 °C/min increase in temperature while thermograms were taken [19].

#### 2.9.3. Thermogravimetric Analysis (TGA)

TG curves were obtained for MCZ, XGM, TXGM, blank, and MCZ-loaded nanoparticles using an alumina crucible and a Shimadzu DTG60 thermobalance set to heat at a rate of 10 °C min^−1^ from 30 to 500 °C, with a dynamic atmosphere of nitrogen at 50 mL min^−1^. The mass of the sample was precisely weighted, weighing approximately 2.5 mg.

#### 2.9.4. X-ray Diffraction Analysis (XRD)

The X-ray diffractometer (PANalytical B.V., Almelo, The Netherlands) was used to measure X-ray diffraction of MCZ, XGM, TXGM, blank and MCZ-loaded nanoparticles. The setup for the generator was 40 kv and 40 Ma. The sample’s reflected radiation was measured at 25 °C and within the 2θ angle ranges of 2° to 80°, using a test speed of 1°/min.

#### 2.9.5. Scanning Electron Microscopy (SEM)

Using SEM, the surface morphology of the particles was investigated. A small conductive layer of gold was applied to the samples for this purpose, and their morphology was then examined under a scanning electron microscope (SEM-JEOL, JSM-7600F, JEOL Inc., Tokyo, Japan) operating at an accelerating voltage of 10–20 kW.

### 2.10. Mucoadhesion Study

The ex vivo adhesion time of TXGM and developed nanoparticles was measured using the intestinal mucosa of sheep as the membrane of biological origin in order to evaluate their mucoadhesive capabilities [20]. Within 30 min of the slaughter, the intestinal mucosa of the sheep was taken and washed with distilled water, followed by a 37 °C simulation of intestinal fluid (pH 6.8). Using cyanoacrylate glue, XGM, TXGM, and developed nanoparticles moistened with one drop of artificial intestinal fluid (pH 6.8), the mucosa was attached on the inside of a beaker. The mucosa was then pasted on using a light fingertip force for a duration of 20 s. Dissolution apparatus type II was used to keep the beaker, which held 900 mL of the simulated intestinal fluid, at 37 °C. Nanoparticle attachment was observed when the liquid was agitated at 100 rpm using a paddle to mimic the intestinal environment. The adhesion time is the time needed for the XGM, TXGM, and developed nanoparticles to separate from the intestinal mucosa. The average mucoadhesion time was obtained by repeating each sample three times (*n* = 3)

### 2.11. Permeation Study

The Franz diffusion cell, which has an interior diameter of 2.5 cm, was used for permeability investigations. As the model membrane, albino rat intestinal mucosa was employed [21]. Dissection was used to get the intestine of the recently dead albino rats. After being carefully removed and clipped from the sides, the intestinal mucosa was cleaned in an isotonic phosphate buffer with a pH of 7.4. In the space between the donor and receptor compartments, the mucosa was mounted. A magnetic bead revolving at 100 rpm was used to stir 30 mL of isotonic phosphate buffer with a pH of 7.4, which was kept at 37 ± 0.5 °C, into the receptor compartment. Following their prior moistening with 2 mL of buffer, the mucosal surface of the membrane came into close contact with the nanoparticles and suspension in water containing 4 mg of MCZ. Samples were taken out at appropriate intervals for up to 24 h, and the HPLC method was used to analyze them after replacing the same amount with fresh media. The measurements of permeability studies from each formulation repeated three times (*n* = 3).

Coefficients of apparent-permeability (*P_app_*, cm/h) and steady state diffusion (*D*, cm^2^/h) were considered using Equations (3) and (4);

(3) $$P_{app}=\frac{J_{SS}}{C_{d}}$$

(4) $$D=J_{app}\times \frac{L}{K}$$

The calculation of *J_ss_* involved graphing the total amount of MCZ permeability per unit area versus time (hours), with the slope of the linear section of the curve representing steady state flux. The initial total donor chamber concentration of MCZ is denoted by *C_d_*. *L* is the diffusion path length, and *K* is the MCZ partition coefficient (logP).

### 2.12. Antifungal Activity of Nanoparticles Containing MCZ

*Candida albicans* (ATCC No. 10231, Sigma-Aldrich Production GmbH, Buchs, Switzerland) was used in the agar-well diffusion method to measure the antifungal activity [22]. For the preparation of inoculum, 24-h-cultures of Candida albicans were used. The concentration of yeast was adjusted to 0.5 McFarland by turbidimetry (Densimat, bioMérieux, Marcy-l’Étoile, France) on saline [23]. This procedure will yield a yeast stock suspension of 1 × 10^6^ to 5 × 10^6^ cells per mL. To prepare 150 mg/mL concentrations of MCZ, NTG1 nanoparticles and MCZ gel are suspended in DMSO along with MCZ, the positive control. In the tests, the DMSO concentration did not go above 0.4%. A typical inoculum of fungal culture was inoculated onto a plate holding Muller-Hinton agar. A, B and C, three 6-mm-diameter wells, were punched into the agar. Wells A and B were filled with the MCZ gel and the prepared MCZ-containing nanoparticles, respectively, and C indicated the control group. The plates were incubated at 37 °C for 48 h. To assess the antifungal activity, the zone of inhibition’s diameter was assessed. Minimum inhibitory concentration (MIC) values of MCZ gel and NTG1 dispersion were measured by making dispersion in DMSO. The lowest concentration that completely stopped yeast growth was identified as the MIC value. Three (*n* = 3) MIC studies were conducted.

### 2.13. In Vitro Cytocompatibility Studies

The MTT assay was used to determine the in vitro cytocompatibility of the synthesized NTG1 nanoparticles in the Caco-2 cell line (ATCC 2112 M St, Washington, DC, USA). MTT assay: a colorimetric examination that relies on live cells’ specific capability to convert MTT into formazan crystals with a purple color [24]. The absorbance maxima of these formazan crystals are located at 570 nm. After dissolving the formed formazan crystals in a solubilization buffer, the absorbance at 570 nm was measured. The quantity of live cells is exactly proportional to the absorbance that was obtained. The percentage of cell viability versus the amount of NTG1 nanoparticles in mg/mL was then shown on a graph. Using particle-free Caco-2 cell culture wells as a negative control, the MTT test was performed at doses varying from 0.2 to 2 mg/mL. The cells and particles were then cultured for 48 h. Next, 10% MTT solution was added, and the mixture was incubated for 4 h. After that, solubilization buffer was added, and the mixture was incubated for an additional hour. With the help of a Beckmann-Coulter Elisa plate reader (Bio-Tek Power Wave XS, Hampton, NH, USA), the absorbance was measured at 570 nm after the MTT had completely dissolved. For every experiment, three duplicate samples were examined.

### 2.14. Stability Study of Nanoparticles

Size, PDI, and surface charge are used to characterize the stability of nanoparticles. Because surface energy and the surface-to-volume ratio both rise at the nanoscale, the stability of nanoparticles is a crucial parameter [25]. Important factors for physical targeting or drug delivery to particular regions are size, PDI, surface charge and %EE. The stability tests were conducted in closed glass vials for 6 months at 4 °C and 25 °C. The size, PDI, surface charge and %EE of NTG1 to NTG5 formulations were repeated three times (*n* = 3).

### 2.15. HPLC Method for MCZ Estimation

The HPLC method was developed for the quantification of MCZ using mobile phase consists of methanol and water in a ratio of 75:25 *v*/*v*. The Hitachi L-2000 series HPLC system, made in Japan, was furnished with a UV-Vis detector L2420 (Leuven, Belgium), L injection loop, and a Model L-2130 pump. A C_18_ column was used for separation with a detection wavelength of 313 nm with 1 mL/min flow rate of used mobile phase. The method was validated via calculating the percentage recoveries, precision, system suitability parameters, limit of detection and quantification.

### 2.16. Pharmacokinetic Analysis

The pharmacokinetics of MCZ was assessed in albino rats weighing between 250–450 g. The twelve albino rats were taken and divided into two groups (*n* = 6). Group 1 (the control group) received a suspension of MCZ in distilled water, and nanoparticles of thiolated XGM containing MCZ were administered to group2 (the test group) orally. After administration, blood samples from the rat tail vein were withdrawn at regular time intervals (0, 0.5, 1, 2, 4, 8, 12, 24, 36, 48 and 72 h) and placed in heparinized tubes. The blood samples were centrifuged at 4500 rpm for 20 min, the plasma was separated, deproteinizer was added and centrifuged again at 4500 rpm for 20 min. The clear supernatant was separated and injected into the HPLC system for analysis. The study determined several pharmacokinetic parameters, including maximum concentration of MCZ in plasma (C_max_), the moment at which plasma concentration peaks appear (t_max_), and area under the plasma concentration–time curve from 0 h to 24 h (AUC_0–t_) and 0 to infinity (AUC_0–∞_), and mean residence time (MRT).

### 2.17. Statistical Analysis

The release pattern of MCZ from nanoparticles was statistically analyzed by one way ANOVA. The student *t* test was also applied to the pharmacokinetics parameters.

## 3. Results and Discussion

### 3.1. Preparation of Nanoparticles and Estimation of Thiol Contents

**Figure 1:**
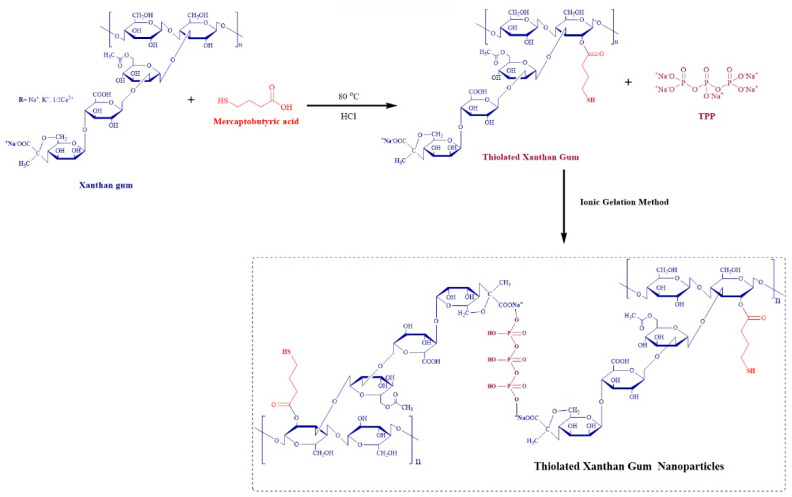
Schematic representation for the formation of TXGM and nanoparticles by ionic gelation method.

The natural gums’ ability to adhere to mucous surfaces can be reinforced by thiolation techniques. In the current study, XGM was thiolated as mentioned in Figure 1, and the synthesized thiomers were then characterized. Using Ellman’s approach, it was found that thiolated XGM had 4.7 mM of thiol contents in 2 mg/mL of TXGM solution.

### 3.2. ^1^H NMR Analysis

**Figure 2:**
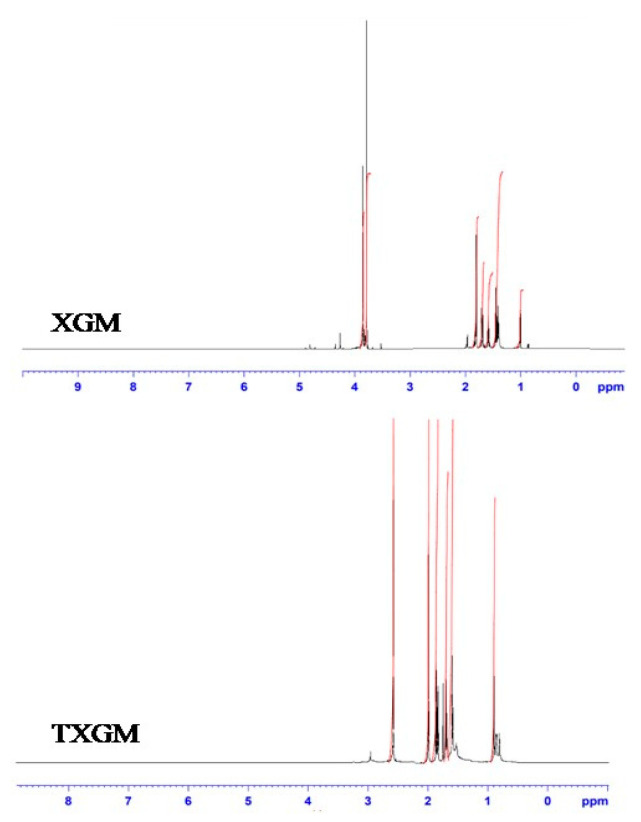
^1^H NMR of XGM and TXGM.

The ^1^H NMR spectra of XGM and TXGM conjugates are shown in Figure 2. The XGM showed distinct peaks at δ 3.9 ppm, δ 1.8 ppm and δ 1.4 ppm due to the sugar protons, acetate group and pyruvate group of XGM [26]. TXGM showed a peak of protons of thiol group at δ 2.5 ppm which confirmed the thiolation of XGM.

### 3.3. Size and Surface Charge Analysis

**Figure 3:**
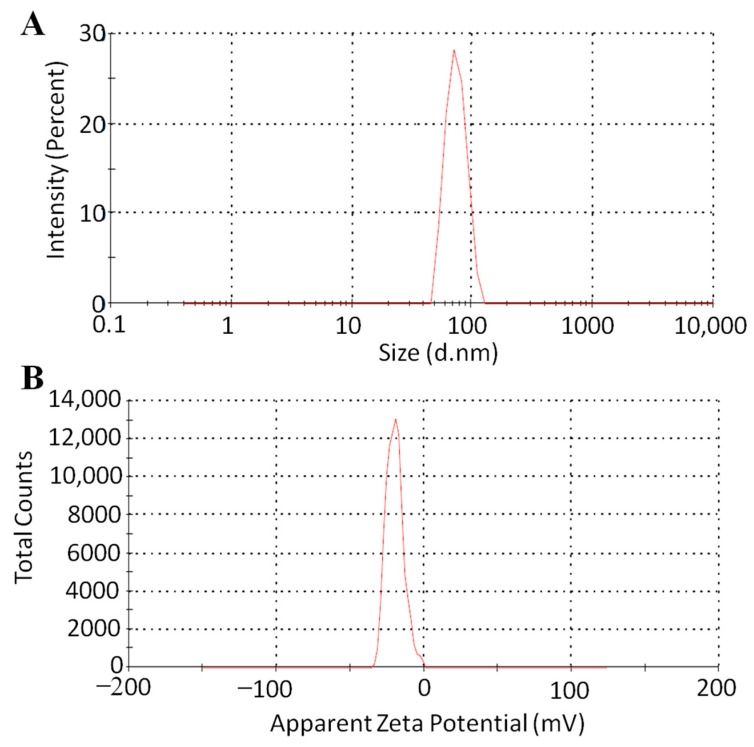
Size (**A**) and surface charge (**B**) of nanoparticles of TXGM of NTG1 formulation.

Figure 3 illustrates the DLS result for thiolated XGM particles, which shows the size distribution of the nanoparticles. The remarkable stability is demonstrated by the fact that functionalized nanoparticles are stable enough in water to conduct the analysis. Colloidal stability in water is a crucial property to prevent aggregation, given the purpose for which the nanoparticles were created. Based on the results for nanoparticles, an average hydrodynamic diameter of 87 ± 2 nm with a polydispersion index (PDI) of 0.32 was determined as shown in Table 1. The research suggests that nanoparticles with sizes between 10 and 200 nm have a higher chance of having increased permeability [27]. Moreover, particles in the 100–200 nm hydrodynamic diameter range are large enough to be retained in the intestinal tract. To gauge the functionalized nanoparticles’ drug affinity, it is also critical to assess the finished nanoparticles’ zeta potential. The tests indicated that the nanoparticles’ zeta potential was −19.9 mV. The functionalized nanoparticles of thiolated XGM have a significant negative charge, which is a direct result of this process. Increased intestinal permeability and residence time are two other benefits of negatively charged nanoparticles [28]. Moreover, the loading of cationic medications, like those examined in this work, depends critically on the negatively charged surface of nanoparticles. Consequently, thiolated XGM nanoparticles have the ideal size to be used as medication nanocarriers for oral delivery [29].

### 3.4. Release and Kinetics of MCZ from Nanoparticles

**Figure 4:**
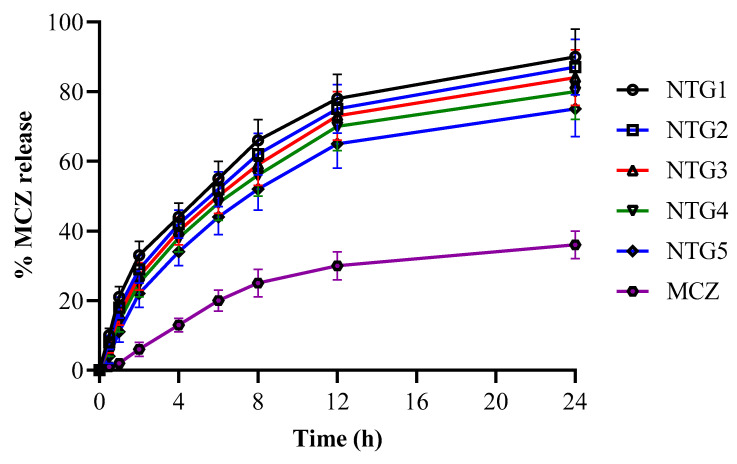
Release of MCZ from nanoparticles of TXGM (NTG1 TO NTG5).

The percent drug loading and entrapment efficiency ranged from 19.5 to 28.5% and 65.5 to 71.5%, respectively. An ideal system where the rate of medication release is within the desired range is ensured by in vitro release analysis. MCZ dispersion and MCZ-containing thiolated XGM nanoparticles were all produced at pH 6.8. The results of this investigation are presented in Figure 4. Within 24 h of the investigation, a 36% release of the MCZ dispersion was seen at pH 6.8, confirming the slow release from its dispersion. After a 24-h experiment, MCZ-containing nanoparticles released 90.5% of the medicine. In contrast to other formulations, these results showed the delayed release behavior of MCZ from nanoparticles, which is an appropriate release rate for an ideal system [30]. The thiolation of XGM was the cause of the extended release from the nanoparticles; the thiolated polymer nanoparticles exhibited mucoadhesion for a long time as a result of the disulfide bond that formed between the sulfur groups of the polymer and mucus [31]. The *p* value was less than 0.001, which indicated that the results were statistically significant.

Based on in vitro drug release data for each formulation, the best matched release kinetic model was identified through modeling of zero-order, first-order, Higuchi, and Korsmeyer–Peppas models. The MCZ release mechanism and best-fit model are validated based on the *R*^2^ and *n* values. The regression coefficient can be found in the *R*^2^ value. When compared to alternative kinetic models, the *R*^2^ values of the Korsmeyer–Peppas model for all formulations of the manufactured hydrogel are closer to 1, indicating that this was the model that suited the data the best. Additionally, the value of “*n*” determines the type of diffusion. It can be observed that the diffusion is non-Fickian when *n* > 0.45 and Fickian when *n* < 0.45. In every formulation, the Fickian diffusion was noted, with *n* values varying between 0.104 and 0.252 as shown in Table 2 [32].

### 3.5. Characterization of Nanoparticles

#### 3.5.1. FTIR

**Figure 5:**
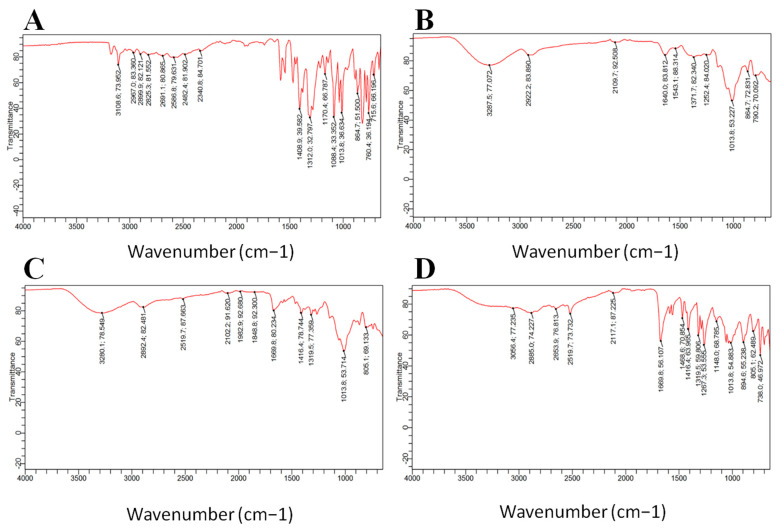
FTIR spectra of MCZ (**A**), XGM (**B**), TXGM (**C**) and nanoparticles of NTG1 (**D**).

FTIR analyses were utilized to evaluate the chemical and physical interactions among MCZ and the excipients utilized in the nanoparticulate formulations. Major peaks for MCZgroups such as stretching of C-N, aromatic stretching at C-H, and aliphatic C-H stretching were seen at 1312 cm^−1^, 3108 cm^−1^, and 2967 cm^−1^, respectively, as shown in Figure 5A. The other notable peaks in MCZ were aromatic C–C bonding at 1408 cm^−1^, C–O stretching at peak 1117 cm^−1^, aromatic out of plane bending at peak 864 cm^−1^, and C–CL stretching at peak 760 cm^−1^ [33]. The FTIR spectra of XGM revealed asymmetric stretching of carboxylate ions by peaks at 1640 cm^−1^ and stretching vibration of primary alcohols C–O at 1013 cm^−1^ [34]. TXGM showed peaks of thiol group at 2519.87 cm^−1^ [12] as shown in Figure 5C. All of the TXGM peaks were present in the NTG1 spectrum, which represents the TPP cross-linked TXGM; however, the intensity of most of them decreased, particularly the 2117 cm^−1^ peak. The peaks of the MCZ were also present in the NTG1 spectra but the intensity was low. This can be explained by the ionic crosslinking reaction between TPP and TXGM, which eventually results in the creation of nanoparticles.

#### 3.5.2. DSC

**Figure 6:**
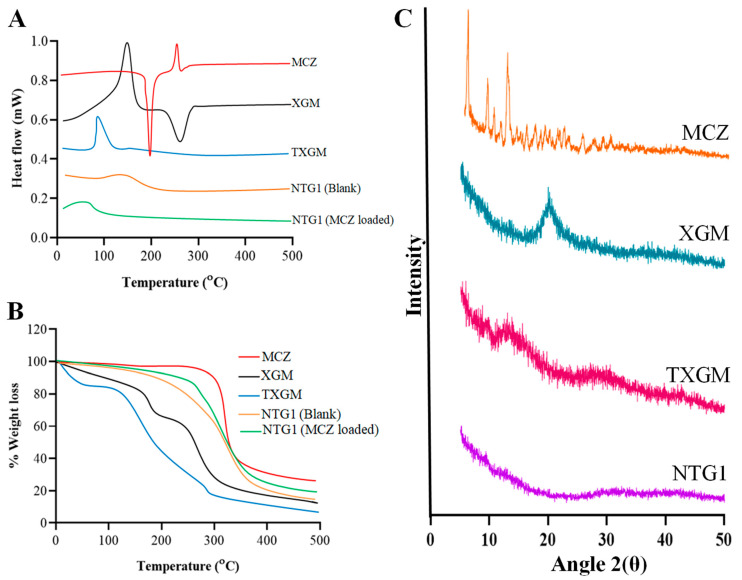
DSC (**A**) and TGA (**B**) of MCZ, XGM, TXGM, blank and MCZ-loaded NTG1 formulation of nanoparticles and XRD (**C**) of MCZ, XGM, TXGM and NTG1 formulation.

Using DSC analysis, the MCZ solubility and physical state in the finished formulation were examined. A prominent endothermic peak of MCZ was visible in the DSC study at 199.5 °C. This is the temperature at which MCZ melts, according todata that havealready been published [35]. The presence of this peak indicated that MCZ was crystalline [36]. A melting endothermic peak is seen in XGM at 151 °C, while a degrading exothermic peak is seen at 277 °C [37]. TXGM showed an endothermic peak at 99 °C, this decrease in melting temperature was due to the easily breakable thiol groups. Figure 6A clearly shows that in the final formulation designated as NTG1, the endothermic peak dissipated. The disappearance of the peak indicates that MCZ has transitioned from a crystalline to an amorphous state that is more soluble. Based on previously published data [38], it may be inferred that the MCZ state was altered to an amorphous form in the final formulation, suggesting high energy and improved solubility results.

#### 3.5.3. TGA

The TGA of MCZ showed thermal stability at 310 °C and 70% weight loss was observed at 312 to 500 °C as shown in Figure 6B. The first reduction in weight of XGM was calculated to be 14.5% because of the moisture and volatile materials that were released at 178.5 °C. Subsequently, there was a two-stage breakdown process: a 49% weight loss in the first stage that took place between 200 and 296 °C, and a 77% weight loss in the second stage that took place between 310 and 500 °C. In contrast to XGM, TXGM degrades at a lower temperature. This makes sense in light of the fact that the presence of readily breakable side chains in the case of thiolated XGM facilitates simpler chain scission. The TPP cross-linking which delays the process of thermal degradation, explains why the breakdown of thiolated XGM nanoparticles is significantly more difficult than that of thiolated XGM itself. The reason for the reduced thermal degradation rate of nanoparticles compared to thiolated XGM is due to the relationship between the various constituents of the polymer, both physically and chemically. Its resistance to thermal degradation was increased as a result of the ionic gelation method creation of nanoparticles. The MCZ-loaded nanoparticles typically broke down at a temperature greater than that of their constituent parts. The earlier discussion led to the conclusion that pure polymers are not as thermally stable as nanoparticles.

#### 3.5.4. XRD and SEM

**Figure 7:**
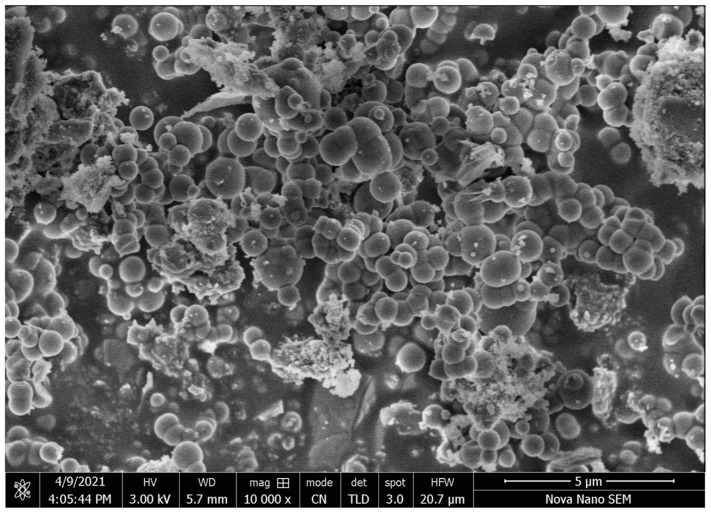
SEM image of MCZ loaded NTG1 formulation of TXGM nanoparticles.

Typically, compounds with peaks that are sharp and intense are referred to as crystalline, whereas compounds with diffuse peaks are referred to as amorphous. The crystalline structure of MCZ is indicated by strong peaks in its diffraction pattern at dispersed angles of around 2θ as shown in Figure 6C. At 2θ = 23.50°, XGM showed a broad, amorphous peak. The XRD of TXGM showed more crystallinity compared to XGM, which was because thiol groups are present. The XRD study of the produced nanoparticles revealed that the ionic gelation process had reduced or eliminated the crystalline and strong peaks of MCZ and TXGM. The findings suggest that the MCZ was successfully entrapped by the nanoparticles, which also displayed decreased crystallinity of the pure drug and low-intensity peaks in place of sharp ones. The SEM images of MCZ-loaded NTG1 showed a spherical shape as mentioned in Figure 7. The difference in particle size measured by SEM is different from the size by DSL method. DLS data are an instrument’s response in relation to particles and is conducted in wet conditions, whereas SEM directly evaluates the size and is carried out in vacuum conditions [39]. Hence SEM might give different particle size contrary to DLS size due to the collision of particles. In DLS, the hydrodynamic diameter is calculated from the diffusion coefficient in set parameters such as solvent density and temperature of measurements which are important, and size is calculated from the detected light fluctuations. Particularly with spherical particles, DLS allows an estimation of the molar particle mass and a description of the size distribution of the particles, while SEM offers an investigation of the morphological appearance of the particles. The charge and concentration of polymers in the formulation and angle dependencies also affect the size of particles measured by DSL and SEM [40].

### 3.6. Mucoadhesion Study

**Figure 8:**
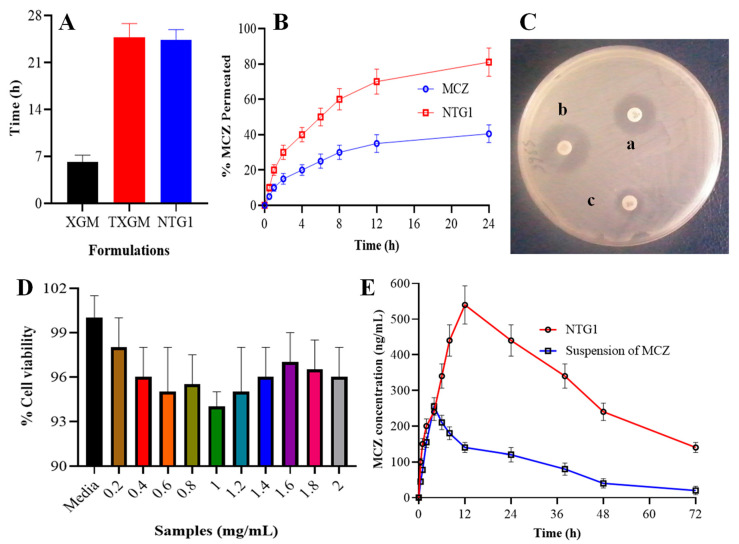
Mucoadhesion of thiolated polymers with nanoparticles of TXGM (**A**), permeation of MCZ from pure MCZ and nanoparticles of NTG1 (**B**), antifungal activity of NTG1 and pure MCZ (**C**), cell viability assay using different concentrations of nanoparticles of NTG1 on Caco-2 cells (**D**) and pharmacokinetics of MCZ from NTG1 and suspension of MCZ (*n* = 6) (**E**). In part (**C**), a, b and c are the zones of inhibition of MCZ gel, NTG1 and control group, respectively.

The time of adhesion with the mucosa varies significantly between XGM and thiolated XGM, according to mucoadhesion experiments. As demonstrated in Figure 8A, the XGM stayed attached to the mucosal membrane for up to 6.2 h, whereas the TXGM and NTG1 totally separated from the membrane after 24.8 and 24.4 h, respectively. Because of the quantity of free thiol groups on the surface of the polymers that form S-S linkages with the cysteine-rich subdomains of the mucus membrane, the adhesion of TXGM was nearly doubled. Because XGM contains the -OH group, which is in charge of non-covalent interactions like hydrogen bonds, van der Waals forces, and ionic interactions, it has also demonstrated mucoadhesion. Strong connection between the polymer and the mucous membrane was demonstrated in the case of TXGM by the addition of all non-covalent interactions of the disulfide bonds because of the presence of the -SH group [41].

### 3.7. Permeation Study

Studies on the amount of MCZ from nanoparticles (NTG1) that permeated the intestinal mucosa of albino rats revealed that it was around 2 times more permeable than the amount of plain drug (MCZ) as shown in Figure 8B. To ascertain the drug’s permeability properties, the ex vivo intestinal mucosal permeation profile of MCZ was fitted to the non-steady state solution of Fick’s second law for a single layer membrane. The quantity of MCZ that permeated 1 cm^2^ of the permeation membrane in a unit of time was measured as the steady state flux (Jss, µg cm^−2^ h^−1^). The calculation of Jss involved graphing the total quantity of MCZ permeabilized per unit area against time (h), with the slope of the linear section of the curve representing steady state flux. It was discovered that the MCZ concentration across the intestinal mucosa was 1.124 ± 0.126 µg cm^−2^ h^−1^ for nanoparticles (NTG1) and 0.610 ± 0.002 µg cm^−2^ h^−1^ for the plain drug (MCZ).

### 3.8. Antifungal Activity of Nanoparticles Containing MCZ

The findings are shown in Figure 8C. Three duplicate readings of each were obtained. It is clear from a comparison of the minimum inhibitory concentration (MIC) values of the two test samples that the NTG1 nanoparticles’ antifungal activity has been significantly increased, which was projected for the formed formulation. Two samples were tested for their antifungal potential using the antifungal assay against Candida albicans, as indicated by the circles in Figure 8C, which was based on ZOI. As shown in Figure 8C, the ZOI for NTG1 nanoparticles and commercial MCZ gel were 40 mm and 25 mm, respectively. These findings showed that NTG1 nanoparticles had 1.8 times the antifungal activity of commercially available MCZ gel. The maximal sensitivity of this gel against Candida albicans was the cause of the maximum ZOI for NTG1 nanoparticles. Additionally, earlier research demonstrated that nanoparticles have a greater capacity to penetrate the fungal cell wall. Consequently, MCZ nanoparticles penetrated the tight connections in the stratum corneum, postponing the synthesis of ergosterol and finally preventing the growth of fungus [22]. The MIC for the NTG1 and MCZ gel formulations is shown in Table 3. We can see from Table 4 that in order to completely prevent the growth of Candida albicans, the dispersion of NTG1 needs a lower concentration of MCZ than MCZ gel. In the typical susceptibility assays, the DMSO may increase the inhibitory activity of medicines that are insoluble in water. The capacity of DMSO to maintain the medication’s greater dispersion and accessibility to suppress yeast growth. More medications can attach to their targets because of the increased plasma membrane fluidity caused by DMSO, which also strengthens the antifungal effects of medications that are water insoluble [42].

### 3.9. In Vitro Cytocompatibility Studies

It was evident from the plot that all concentrations of the NPs samples under investigation did not significantly differ in terms of percentage cell viability. Likewise, we contrasted the NPs’ level of toxicity with that of the negative control. As can be seen from the image, about 95% of the cells are viable at all of the NTG1 nanoparticle concentrations when compared to the negative control (Figure 8D). The produced nanoparticles appear to be less hazardous to colon cancer cells, which is a necessary condition for their usage in biomedical applications, according to these studies [8].

### 3.10. Stability of Nanoparticles Containing MCZ

The assessment of the stability of polymeric nanoparticles loaded with MCZ was conducted by evaluating the drug encapsulation efficiency, particle size, PDI, and zeta potential of the initial preparations with those that were kept in covered glass vials for 6 months at 25 °C and 4 °C. When these values were kept at 4 °C, no appreciable changes were seen. However, as Table 4 indicates, there were notable modifications to the physicochemical characteristics when the product was held at 25 °C. These alterations could be related to the clumping of particles during extended storage; however, at low temperatures, the low kinetic energy of the particles inhibits the clustering of particles and hence prevents particle collisions. Based on these findings, nanoformulations should be stored at 4 °C to ensure optimal efficacy in therapy and to avoid physical as well as chemical instability [25].

### 3.11. HPLC Method for MCZ Estimation

With the possible exception of a minor adjustment to the mobile phase, the chromatographic conditions were chosen to be consistent with those specified in the USP. The devised method is sensitive and selective enough to be employed for quantitative potency analysis, according to the results of the analytical method validation. The percentage average standard deviation of six replicates of the standard solution was 0.13%, indicating that the system was found to be reproducible. The correlation coefficient (r) was 0.9993. This linearity was found to be between 3.125 and 50 µg/mL. Since there was no evidence of interference between MCZ and the other product excipients, the approach was deemed to be selective [43]. The validated parameters were calculated and mentioned in Table 5. The recoveries of MCZ ranged from 97.60 to 90.08% and the percentage RSD was less than 2 in all parameters. The retention time of MCZ was 6.8 min. Limit of detection and quantification were 0.14 and 0.19 µg/mL, respectively.

### 3.12. Pharmacokinetic Analysis

NTG1 was used in the in vivo investigations because it offered a regulated release pattern for the medication. Figure 8E and Table 6 illustrate the mean plasma concentration–time data of MCZ after NTG1 and the control were administered orally. NTG1 had a mean peak plasma concentration (C_max_) of 540 ng/mL and a mean tmax of 12 h, whereas the control group had a C_max_ of 255 ng/mL and a mean t_max_ of 4 h showed 1-fold increase in C_max_ and 2-fold increase in t_max_ as shown in Figure 8E. This might be because thiolated XGM nanoparticles have a longer mucoadhesion time. The longer mucoadhesion time leads to the enhanced permeation which ultimately enhanced the pharmacokinetics of MCZ from nanoparticles [44]. The thiolated XGM effectively lengthens the nanoparticles time of adherence to the mucosa. The results showed that the control and NTG1 had mean AUC_0–t_ values of 2359 and 3245 ng.h/mL, respectively. The values of AUC of MCZ from nanoparticles weregreater the control formulation [45]. A 6-fold increase in MRT of MCZ from NTG1 was observed compared to the control group.

## 4. Conclusions

TXGM, a mucoadhesive polymer, was synthesized and examined. Using Ellman’s procedure, the degree of thiol substitution was determined to be 61%. FTIR, DSC, TGA, XRD, SEM, and DLS analyses were used to characterize the synthesized TXGM nanoparticles. The produced nanoparticles have a spherical shape and range in size from 87 to 156 nm. Zeta potential measurements were used to study the system’s stability. It became apparent that the nanoparticles had a negative surface charge, and that the system was remarkably stable. Using the MTT assay, the produced nanoparticles’ in vitro cytocompatibility with cancer cell lines was investigated. According to these findings, the produced nanoparticles are less harmful (almost 93% of the cells remain viable in the TXGM nanoparticle samples at all dosages). Therefore, the produced TXGM nanoparticles can be applied as a useful biomaterial in biomedical applications including gene and mucoadhesive medication delivery.

## Figures and Tables

**Table 1:** Composition of nanoparticles, size, PDI and surface charge of nanoparticles of TXGM.

Code	Composition of Formulations (Ratio of TXGM and STPP)		Size (nm)	PDI	Zeta Potential (mV)	Loading of MCZ (%)	Entrapment Efficiency (%)
	TXGM	STPP					
NTG1	1	1	87 ± 2	0.23 ± 0.02	−17 ± 2	28.5 ± 2.52	71.5 ± 3.56
NTG2	1	2	98 ± 3	0.39 ± 0.03	−15 ± 3	25.4 ± 1.80	69.8 ± 3.08
NTG3	1	3	121 ± 2	1.09 ± 0.03	−11 ± 2	23.2 ± 1.62	67.4 ± 4.01
NTG4	1	4	134 ± 5	1.23 ± 0.05	−08 ± 4	21.8 ± 1.02	66.2 ± 3.98
NTG5	1	5	156 ± 6	0.82 ± 0.02	−07 ± 2	19.5 ± 1.42	65.5 ± 4.28

NTG1 to NTG5 are the nanoparticles of thiolated xanthan gum.

**Table 2:** Kinetics of MCZ from nanoparticles prepared by ionic gelation method.

Code	Zero Order	First Order	Higuchi	Korsmeyer-Peppas	
	*R* ^2^	*R* ^2^	*R* ^2^	*R* ^2^	*n*
NTG1	0.999	0.564	0.991	0.999	0.104
NTG2	0.997	0.487	0.990	0.992	0.209
NTG3	0.996	0.509	0.878	0.997	0.252
NTG4	0.998	0.498	0.894	0.998	0.179
NTG5	0.999	0.645	0.993	0.999	0.250

**Table 3:** MIC values of MCZ gel and NTG1 dispersions (Dissolved in DMSO).

Formulations	MIC (µg/mL)
MCZ gel	4.01 ± 0.34
NTG1	1.34 ± 0.21

**Table 4:** Stability studies of nanoparticles of thiolated XGM containing MCZ.

Duration	Code	At 4 °C				At 25 °C			
		Size (nm)	PDI	Zeta Potential (mV)	% EE	Size (nm)	PDI	Zeta Potential (mV)	% EE
1 month	NTG1	88 ± 3	0.24 ± 0.02	−16 ± 2	69 ± 2	91 ± 3	0.69 ± 0.02	−11 ± 2	55 ± 3
	NTG2	99 ± 4	0.39 ± 0.03	−15 ± 4	65 ± 3	112 ± 4	0.98 ± 0.03	−09 ± 3	51 ± 2
	NTG3	122 ± 2	1.19 ± 0.03	−10 ± 1	64 ± 2	145 ± 3	1.76 ± 0.03	−04 ± 2	45 ± 3
	NTG4	135 ± 4	1.26 ± 0.06	−08 ± 3	62 ± 2	176 ± 4	1.92 ± 0.05	−06 ± 4	43 ± 2
	NTG5	158 ± 5	0.84 ± 0.03	−08 ± 2	59 ± 3	198 ± 7	1.42 ± 0.02	−05 ± 2	39 ± 3
3 month	NTG1	91 ± 4	0.24 ± 0.03	−16 ± 2	67 ± 3	95 ± 4	0.80 ± 0.03	−05 ± 1	51 ± 2
	NTG2	100 ± 5	0.40 ± 0.02	−14 ± 3	63 ± 4	117 ± 3	1.10 ± 0.04	−04 ± 2	43 ± 2
	NTG3	124 ± 4	1.20 ± 0.04	−10 ± 3	61 ± 3	149 ± 4	1.89 ± 0.06	−02 ± 2	38 ± 3
	NTG4	136 ± 6	1.26 ± 0.05	−08 ± 2	56 ± 4	181 ± 5	2.23 ± 0.04	−01 ± 3	35 ± 2
	NTG5	159 ± 4	0.85 ± 0.06	−07 ± 2	54 ± 2	201 ± 6	2.46 ± 0.06	01 ± 1	31 ± 2
6 month	NTG1	95 ± 5	0.25 ± 0.05	−15 ± 3	64 ± 3	109 ± 5	0.87 ± 0.03	−03 ± 1	44 ± 2
	NTG2	101 ± 3	0.40 ± 0.06	−13 ± 3	56 ± 4	134 ± 4	1.67 ± 0.06	−01 ± 1	41 ± 2
	NTG3	125 ± 4	1.21 ± 0.07	−09 ± 4	55 ± 2	159 ± 5	2.59 ± 0.07	04 ± 2	33 ± 3
	NTG4	137 ± 6	1.27 ± 0.05	−07 ± 2	51 ± 3	189 ± 5	2.83 ± 0.05	06 ± 2	30 ± 2
	NTG5	160 ± 7	0.85 ± 0.07	−06 ± 5	48 ± 2	214 ± 6	2.96 ± 0.04	07 ± 2	26 ± 2

**Table 5:** Percentage recoveries, precision and suitability parameters of developed method of MCZ.

Percentage Recovery of MCZ				Inter-Day Precision		Intra-Day Precision	
Injected Concentration (µg/mL)	Recovered Concentration (µg/mL)	%RSD	Recovery (%)	Mean ± SD (µg/mL)	%RSD	Mean ± SD (µg/mL)	%RSD
50.00	48.12 ± 1.23	0.24	96.24	48.14 ± 2.10	0.29	48.16 ± 1.98	0.32
25.00	23.22 ± 1.90	0.56	92.88	23.24 ± 1.03	0.59	23.25 ± 0.97	0.60
12.50	11.26 ± 2.09	0.67	90.08	11.27 ± 1.23	0.71	11.28 ± 1.02	0.75
6.25	6.10 ± 1.09	0.22	97.60	6.13 ± 0.87	0.24	6.11 ± 0.46	0.23
3.125	2.99 ± 0.97	0.31	95.68	3.01 ± 0.73	0.42	3.03 ± 0.54	0.41
System suitability parameters							
Parameters		Mean (*n* = 5)		%RSD		Limits	
Peak area		14,356		1.34		<2	
Retention time in min		6.8		0.98		--	
Theoretical plates		8239		0.67		--	
Resolution		9.87		1.54		>1	
Tailing factor		1.78		0.91		<2	

**Table 6:** Parameters of pharmacokinetics of MCZ from NTG1 (test) and suspension of MCZ (control) in albino rats (*n* = 6).

Parameters	Units	NTGI (Test)	Suspension of MCZ (Control)
C_max_	ng/mL	540 ± 24 *	255 ± 13 *
t_max_	Hour	12 ± 2 **	4 ± 1 *
t_1/2_	Hour	24.5 ± 5 **	8.5 ± 3 **
AUC_0–t_	ng.h/mL	3245 ± 45 ***	2359 ± 33 *
AUC_0–ꝏ_	ng.h/mL	3654 ± 65 **	2653 ± 32 **
MRT	Hour	47.5 ± 6 **	8.5 ± 2 *

* *p* value is <0.05, ** *p* value is <0.01 and *** *p* value <0.001.

## Data Availability

“MDPI Research Data Policies” at https://www.mdpi.com/ethics, accessed on 17 December 2023.
